# Modulation of the Mechanisms Driving Transthyretin Amyloidosis

**DOI:** 10.3389/fnmol.2020.592644

**Published:** 2020-12-11

**Authors:** Filipa Bezerra, Maria João Saraiva, Maria Rosário Almeida

**Affiliations:** ^1^Molecular Neurobiology Group, IBMC—Instituto de Biologia Molecular e Celular, i3S—Instituto de Investigação e Inovação em Saúde, Porto, Portugal; ^2^Department of Molecular Biology, ICBAS—Instituto de Ciências Biomédicas Abel Salazar, Universidade do Porto, Porto, Portugal

**Keywords:** transthyretin, amyloidosis, mechanism of disease, therapy, amyloid inhibitors

## Abstract

Transthyretin (TTR) amyloidoses are systemic diseases associated with TTR aggregation and extracellular deposition in tissues as amyloid. The most frequent and severe forms of the disease are hereditary and associated with amino acid substitutions in the protein due to single point mutations in the *TTR* gene (ATTRv amyloidosis). However, the wild type TTR (TTR wt) has an intrinsic amyloidogenic potential that, in particular altered physiologic conditions and aging, leads to TTR aggregation in people over 80 years old being responsible for the non-hereditary ATTRwt amyloidosis. In normal physiologic conditions TTR wt occurs as a tetramer of identical subunits forming a central hydrophobic channel where small molecules can bind as is the case of the natural ligand thyroxine (T_4_). However, the TTR amyloidogenic variants present decreased stability, and in particular conditions, dissociate into partially misfolded monomers that aggregate and polymerize as amyloid fibrils. Therefore, therapeutic strategies for these amyloidoses may target different steps in the disease process such as decrease of variant TTR (TTRv) in plasma, stabilization of TTR, inhibition of TTR aggregation and polymerization or disruption of the preformed fibrils. While strategies aiming decrease of the mutated TTR involve mainly genetic approaches, either by liver transplant or the more recent technologies using specific oligonucleotides or silencing RNA, the other steps of the amyloidogenic cascade might be impaired by pharmacologic compounds, namely, TTR stabilizers, inhibitors of aggregation and amyloid disruptors. Modulation of different steps involved in the mechanism of ATTR amyloidosis and compounds proposed as pharmacologic agents to treat TTR amyloidosis will be reviewed and discussed.

## Introduction

Amyloidosis comprises a group of diseases which are characterized by extracellular deposition of protein aggregates, with a structure mainly composed of cross β-sheets, insoluble and toxic, in a range of tissues leading to the dysfunction of normal surrounding tissue (Galant et al., 2017). This review summarizes the current knowledge concerning TTR amyloidosis (ATTR amyloidosis) modulation aiming therapy and discusses the influence of other processes and factors such proteolysis and extracellular chaperones, respectively, on TTR amyloidogenesis in order to contribute for better understanding the disease pathophysiology and for the development of new therapeutic approaches.

## Transthyretin (TTR) Structure and Function

Transthyretin (TTR) is a 55 kDa homotetrameric globular protein constituted by four monomers of 127 amino acid residues (Kanda et al., 1974). It is mainly produced by the liver and choroid plexus of the brain, being then secreted into the blood and cerebrospinal fluid (CSF), respectively. However, TTR synthesis has also been described in other tissues, such as the retinal pigment epithelia (RPE), a monolayer of cells acting as a blood barrier for the retina, which in turn secretes TTR to the vitreous humor (Richardson, 2009). Low levels of TTR expression were also found in Schwann cells of the sciatic nerve, as described by Murakami et al. (2010).

TTR structure was firstly determined in the seventies by Blake and collaborators (Blake et al., 1978), who described that each TTR monomer is organized into two four-stranded anti-parallel β-sheets (A through H) and a short β-helix located on β-strand E (Blake et al., 1978); two monomers are connected through hydrogen bonds between the two H strands of neighboring monomers resulting in a very stable dimer. The association of two dimers, mainly through hydrophobic interactions between residues of the AB to GH loops results in the formation of the TTR tetramer (Blake et al., 1978; Yokoyama et al., 2012).

**Figure 1:**
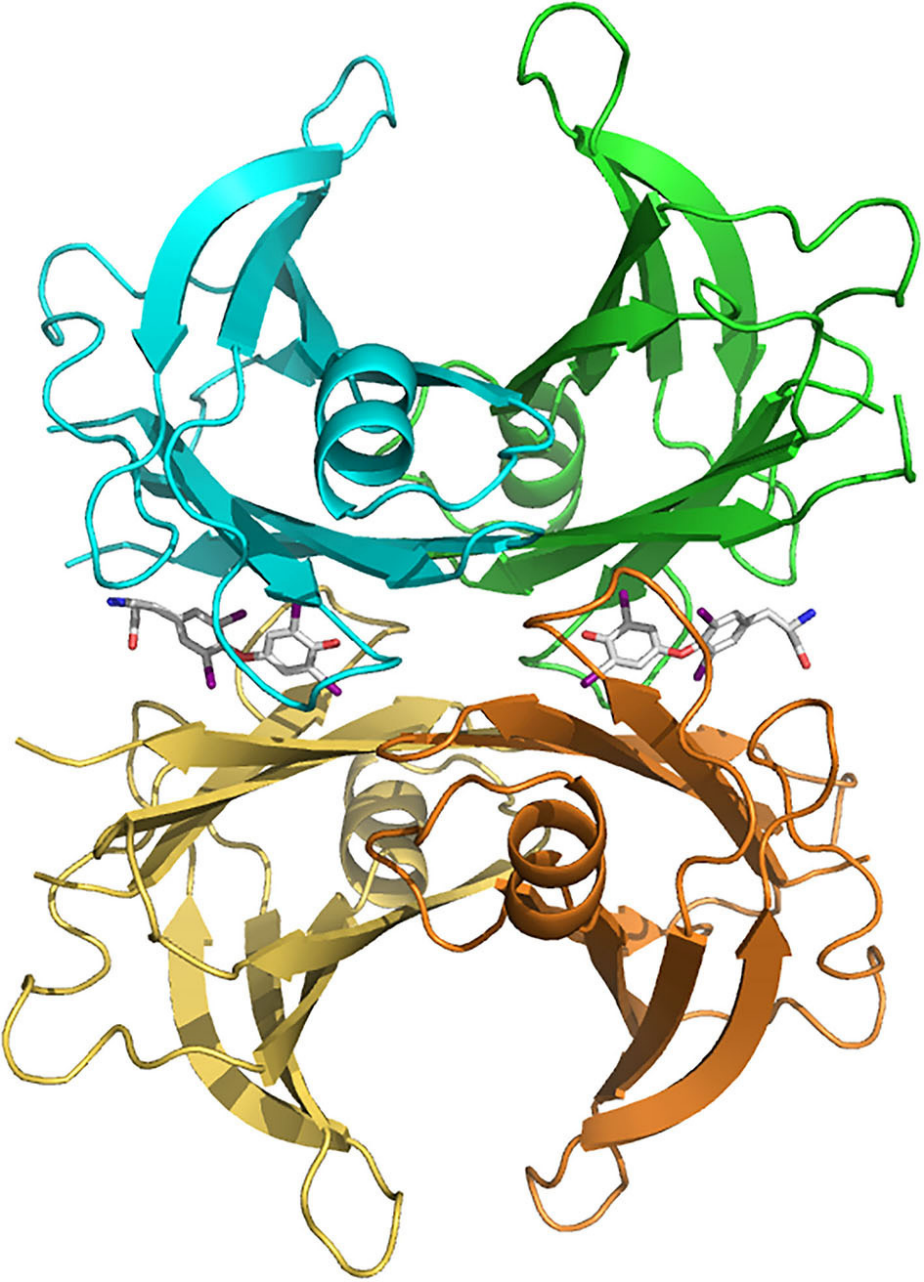
Human transthyretin (TTR) tetramer structure in complex with two thyroxine molecules (stick models) bound in the central hydrophobic channel, from PDB 2ROX (Wojtczak et al., 1996). TTR subunits are colored differently (blue, green, brown and yellow). Figure made with PyMOL (DeLano, 2005).

TTR mainly functions as a carrier protein (Buxbaum and Reixach, 2009; Vieira and Saraiva, 2014). The homotetrameric structure of native TTR forms a central hydrophobic channel that harbors two thyroxine (T_4_) binding sites at the dimer-dimer interface (Blake et al., 1974) (Figure 1). However, due to negative cooperativity, only one molecule of T_4_ is transported by TTR (Andrea et al., 1980). In humans, around 15% of plasma T_4_ is transported by TTR, whereas in rodents this percentage increases to 50% (Vieira and Saraiva, 2014). In the CSF, TTR is the major carrier of T_4_, transporting around 80% of the hormone in both humans and rodents (Hu et al., 2006), being recently described as essential for the retention of T_4_ in the CSF (Chen et al., 2016).

The TTR tetramer has four additional binding sites at the protein's surface for retinol-binding protein (RBP), two in each dimer. Due to steric hindrance, only two RBP molecules may effectively bind to TTR but, since the RBP levels in plasma are lower than TTR, only one RBP molecule is effectively bound to the TTR tetramer (Folli et al., 2010). The assembly of this TTR-RBP complex is essential for the transport of retinol (or vitamin A), allowing its delivery to the cells (Raghu and Sivakumar, 2004). Indeed, studies in TTR knockout mice revealed a decrease in both retinol and RBP levels in plasma (van Bennekum et al., 2001), as well as an accumulation of hepatic RBP (Wei et al., 1995), comparatively to wild-type mice. Altogether these results suggest the pivotal role of TTR as a carrier of the retinol-RBP complex preventing its glomerular filtration by the kidney (Wei et al., 1995; van Bennekum et al., 2001; Gaetani et al., 2002).

Besides its functions as carrier protein, a proteolytic activity has been attributed to TTR. A small fraction of plasma TTR (1–2%) was found associated with high density lipoproteins (HDL) via apolipoprotein AI (apoA-I) (Sousa et al., 2000) and, later, the capacity of TTR to cleave apoA-I carboxyl terminal domain *in vitro* was also demonstrated (Liz et al., 2004). In addition to apoA-I, TTR is also involved on the cleavage of both neuropeptide Y (NPY) (Liz et al., 2009) and Aβ peptide (Costa et al., 2008), suggesting an important role of TTR-mediated proteolysis either in physiologic or pathologic conditions, with major impacts on the biology of nervous system and Alzheimer's disease, respectively (Liz et al., 2010).

A neuroprotective role of TTR has also been described under conditions of cerebral ischemia in mice deficient for heat shock transcription factor 1 (HSF1), an activator of heat-shock proteins. Under conditions of compromised heat-shock response, TTR from CSF contributes to control neuronal cell death, edema and inflammation, thereby influencing the survival of endangered neurons in cerebral ischemia (Santos et al., 2010). More recent work, indicates that TTR acts as neurotrophic factor, through interaction with megalin, stimulating neurite outgrowth and promoting neuroprotection in ischemic conditions (Gomes et al., 2016, 2019).

## Transthyretin-Related Amyloidosis

Transthyretin amyloidosis (ATTR amyloidosis) is a group of diseases in which TTR variants (ATTRv) or even the wild-type protein (ATTRwt) aggregate and form amyloid fibrils that deposit extracellularly in tissues (Sipe et al., 2016). These are, respectively, hereditary and non-hereditary forms of the disease. The non-hereditary form is related to alterations of environmental conditions and aging leading to the aggregation and fibril formation of wild type TTR, ATTRwt (Sipe et al., 2016). Thus, ATTRwt amyloidosis is mainly an age-related disorder, affecting 12–25% of the population over 80 years-old and is characterized by ATTRwt deposition, particularly in the heart, affecting cardiac functions (Westermark et al., 2003). In contrast, the hereditary forms of the disease, ATTRv amyloidosis result from single point mutations in the coding region of the *TTR* gene, mainly producing less stable variant proteins with an altered amino acid in the polypeptide chain, ATTRv (Saraiva, 1995). Accordingly, to date, more than 140 mutations on the *TTR* gene have been described (http://amyloidosismutations.com/mut-attr.php) (Connors et al., 2003). Among these, only about 15 TTR variants are reported as non-amyloidogenic, while most TTR point mutations induce systemic amyloidosis with predominant neuropathic (Plante-Bordeneuve and Said, 2011), or cardiac phenotypes (Rapezzi et al., 2010). However, most of the variants have been associated with a mixed phenotype, characterized by varying degrees of neurological and cardiac involvement (Conceicao et al., 2019). Less frequently, manifestations of ATTR amyloidosis include vitreous opacities (Ando et al., 1997) and, in rare cases, leptomeningeal amyloidosis (Maia et al., 2015).

The substitution of valine for methionine at position 30 (V30M) in the TTR polypeptide chain was the first mutation to be identified and, is the most common mutation associated with ATTR polyneuropathy (ATTR-PN) (previously designated familial amyloid polyneuropathy—FAP) (Saraiva et al., 1984). This life-threatening disease, first described by Corino de Andrade (Andrade, 1952) mainly affects both peripheral and autonomic nervous system, being sensorimotor polyneuropathy, autonomic dysfunction and gastrointestinal tract disturbances the major clinical manifestations which may lead to death within 10 years after disease onset if not treated (Ando et al., 2005; Conceicao et al., 2016).

The prevalence of ATTR V30M amyloidosis is estimated to be 0.87–1.1 per 1 000 000 individuals (Adams et al., 2014) and the disease has been considered endemic in the north of Portugal (Sousa et al., 1995), Japan (Kato-Motozaki et al., 2008), and Sweden (Sousa et al., 1993). Individuals from Portugal and some provinces in Japan typically manifest early-onset and high-penetrance phenotype, whereas people in Sweden and, also in other Japanese regions usually present late-onset and low-penetrance disease (Plante-Bordeneuve and Said, 2011).

Besides peripheral neuropathy, cardiomyopathy is also one of the major clinical manifestations of ATTR amyloidosis (ATTR-CM) (Suhr et al., 2003). In addition to ATTRwt, which is the main cause of ATTR-CM, as mentioned above, some non-V30M mutations on *TTR* gene also lead to the development of cardiac symptoms (Westermark et al., 1990). In particular, TTR V122I is the most common variant responsible for ATTR-CM being almost exclusively found in 3–4% of African-Americans and, the predominant phenotype associated with this mutation is severe restrictive cardiomyopathy with late-onset, i.e., occurs mainly after the age of 60, without neurological symptoms (Jacobson et al., 1997; Quarta et al., 2015; Buxbaum and Ruberg, 2017). There are also other TTR variants responsible for the development of cardiac amyloidosis, such as T49A, S50I, T60A, I68L, and L111M (Rapezzi et al., 2015; Sekijima, 2015).

In patients with ATTR-CM, amyloid fibrils can infiltrate any or all cardiovascular structures including conduction system, the atrial and ventricular myocardium, valvular tissue and, the coronary and large arteries (Falk and Dubrey, 2010). Myocardial infiltration results in progressive increase in the thickness of left and right ventricular walls and of the interatrial septum, ultimately leading to heart failure (Rapezzi et al., 2010).

The diagnosis of ATTR-CM firstly includes echocardiogram and electrocardiogram (Donnelly and Hanna, 2017). However, myocardial scintigraphy using bone avid tracers, in particular, technetium-based isotypes, such as 99^m^technetium 3,3-diphosphono-1,2-propanodicarboxylic acid, pyrophosphate and hydroxymethylene diphosphonate revealed high sensitivity and specificity to cardiac ATTR amyloid deposits. In fact, these agents allow to identify deposits before increasing myocardial wall thickness, contributing to early diagnosis of ATTR-CM (Maurer et al., 2019). In addition, alterations in the values of cardiac biomarkers have also been increasingly helpful on the management of ATTR-CM. Indeed, clinical data from Patel and Hawkins indicate that substantial ATTR amyloid deposits accumulating in the cardiac tissue are accompanied by a moderate increase in serum levels of NT-proBNP concentration (Patel and Hawkins, 2015).

Approximately, one-fourth of the amyloidogenic *TTR* mutations originates vitreous amyloid, namely F33I, R34G, L35T, I84S, and T114C (Sekijima, 2015). It has been postulated that vitreous amyloid is the result of local TTR synthesis in the RPE cells in the eye (Ando et al., 1997). Similarly to vitreous, also leptomeningeal amyloidosis may be related to local TTR synthesis, in this case by choroid plexus and, amyloid deposition mainly occurs in the media and adventitia of medium-sized and small arteries, arterioles and veins of the cortex and leptomeninges. These amyloid infiltrations induce cerebral infarction, cerebral hemorrhage, subarachnoid hemorrhage and hydrocephalus, ultimately leading to serious central nervous dysfunctions, namely ataxia and dementia (Maia et al., 2015; Sekijima, 2015). Till now, leptomeningeal amyloidosis is mainly associated with D18G, A25T, and T114C TTR mutations (Sekijima, 2015). However, in some cases, leptomeningeal amyloidosis may also develop in patients with V30M mutation (Maia et al., 2015).

## TTR Amyloid Formation

The hallmark of ATTR amyloidosis is the extracellular deposition of aggregated TTR or TTR fibrils in tissues. The process of TTR aggregation and fibril formation is not completely elucidated however biochemical and biophysical evidences indicate that the tetrameric form of TTR becomes unstable and the protein dissociates into dimers and monomers presenting a partially unfolded conformation which self-assemble into toxic non-fibrillar aggregates and, later into amyloid fibrils that accumulate as amyloid deposits throughout the body (Quintas et al., 2001; Cardoso et al., 2002).

*In vivo* the amyloid deposits are composed also by other proteins such as serum amyloid P component (SAP) and proteoglycans (Benson et al., 2018a). In the case of ATTR amyloidosis, TTR in the amyloid deposits might be in its intact form meaning as full-length protein and as TTR fragments suggesting that proteolysis might contribute as a mechanism of amyloid formation.

Knowledge of the mechanisms involved in TTR amyloid formation allows establishing therapeutic targets to avoid and/or halt the progression of disease. In this sense several therapeutic strategies have been pursued targeting different stages of the process of amyloid formation or clearance of pre-formed fibrils. The main targets have been lowering or silencing TTR, TTR stabilization, inhibition of TTR fibril formation and fibril disruption that will be discussed below.

## ATTR Amyloidosis Therapies Targeting TTR Synthesis

TTR variants are the main component of amyloid deposits in ATTRv amyloidosis, therefore abolishment of TTR synthesis was one of the first proposed therapeutic approaches in these diseases. Since the liver is the main organ producing and secreting TTR into blood, liver transplant emerged as a possible therapeutic strategy for ATTR amyloidosis (Lewis et al., 1994). Indeed, orthotopic liver transplant (OLT) was shown to arrest disease progression through suppression of mutant TTR from circulation and has been the most effective treatment for ATTR amyloidosis (Benson, 2013; Ericzon et al., 2015). Despite the favorable prognosis observed in transplanted patients, there are still some concerns and long-term complications since the previously existing hereditary amyloid deposits may recruit newly circulating TTR wt promoting amyloid growth and, ultimately resulting in disease progression (Maurer et al., 2016; Saelices et al., 2018). Additionally, there are reports of continuous amyloid deposition even after liver transplantation, mainly in cardiac tissue of transplanted TTR V30M carriers (Okamoto et al., 2011), as well as in the vitreous humor (Munar-Ques et al., 2000; Ando et al., 2001) and leptomeninges (Sekijima et al., 2016). This may be due to the recruitment of newly circulating TTR wt by the previously existing amyloid deposits, mainly in the heart or due to the local TTR synthesis in the eye and choroid plexus of the brain, in either case this might result in amyloid growth and disease progression (Maurer et al., 2016; Saelices et al., 2018). On the other hand, in the case of domino liver transplant (DLT), in which the liver excised from ATTRv patient is transplanted to patients with severe liver disease, the recipients developed symptoms related to ATTR amyloidosis (Stangou et al., 2005; Goto et al., 2006; Barreiros et al., 2010). In addition, post-mortem analysis indicated that systemic amyloid deposition occurred before the appearance of the symptoms (Koike et al., 2011). Interestingly, the clinical manifestations of acquired ATTR amyloidosis after DLT are predominantly related to sensory deficits contrary to the predominant autonomic symptoms in the donors (Stangou et al., 2005; Goto et al., 2006; Barreiros et al., 2010).

The previous findings of continuous amyloid deposition, even after OLT, as well as the knowledge that TTR wt may also aggregate into amyloid fibrils (Westermark et al., 2003), led to an increasing interest in less invasive treatments aiming also to arrest TTR synthesis through gene silencing as a new strategy for the treatment of ATTR amyloidosis. Two different gene-silencing approaches have been developed. One is based on antisense nucleotides (ASOs) and the other on small-interfering RNA (siRNA) (Gertz et al., 2019).

TTR specific siRNAs were firstly tested in mouse models of ATTR amyloidosis and a reduction on amyloid deposition was observed, inducing ATTR amyloid regression (Butler et al., 2016). Different siRNAs with similar mechanism of action were further assessed, in particular patisiran and revusiran, being patisiran the one selected for phase III clinical trials (Coelho et al., 2013). Prolonged administration of patisiran in an open-label study for an extended period of 29 months demonstrated a consistent lowering of plasma TTR levels resulting in disease stabilization and absence of major safety concerns, confirming its indication for ATTR amyloidosis therapy (Adams et al., 2018).

More recently, a novel liver-directed siRNA conjugate, vutrisiran, has been formulated. Vutrisiran is a GalNAc-siRNA conjugate presenting improved pharmacokinetic and pharmacodynamic properties allowing potent and sustained TTR reduction and an acceptable safety profile with mild treatment-related adverse effects as found in a phase I clinical trial enrolling healthy individuals (Habtemariam et al., 2020). These improved characteristics suggest vutrisiran as a novel promising therapy for the treatment of ATTR amyloidosis.

Furthermore, a second-generation ASOs (e.g., IONIS-TTRRx or Inotersen) was reported to be effective by decreasing TTR plasma levels in both monkeys and ATTR I84S transgenic mice. Experiments in healthy humans also revealed a decrease in TTR wt plasma concentrations in a dose-dependent manner (Ackermann et al., 2016). More recent results from phase 3 clinical trial studies with these two gene silencers, patisiran and inotersen, reveal that both are able to efficiently reduce TTR synthesis and arrest disease progression though with some differences in the form and frequency of the therapeutic administration and safety monitoring (Adams et al., 2018; Benson et al., 2018b; Gertz et al., 2019; Koike and Katsuno, 2020).

## ATTR Amyloidosis Therapies Targeting Amyloid Formation

Several compounds have been suggested for the treatment of ATTR amyloidosis by targeting different steps of the amyloid formation. The main steps include TTR stabilization, inhibition of oligomerization and fibril disruption. The most relevant compounds are listed in Table 1 and will be discussed in the following sections. In the recent years, computational studies, such as molecular dynamics (MD) simulations, molecular docking and quantitative structural-activity relationships (SAR) have been used as complement of experimental approaches to better understand TTR monomer misfolding mechanisms-driving TTR amyloidogenesis (Zhou et al., 2019), as well as the binding of small molecules to TTR (Dessi et al., 2020). Indeed, these *in silico* experiments have been essential to obtain a more detailed information about structural changes in biomolecules and, have been used to determine the structural dynamics of TTR (Ortore and Martinelli, 2012; Zhao and Lei, 2014), which in turn will be particularly relevant to the development of more targeted and effective therapies for the treatment of ATTR amyloidosis.

**Table 1:** Compounds proposed for the treatment of ATTR amyloidosis.

**Compound/structure**	**Activity**	**References**
**DIFLUNISAL** 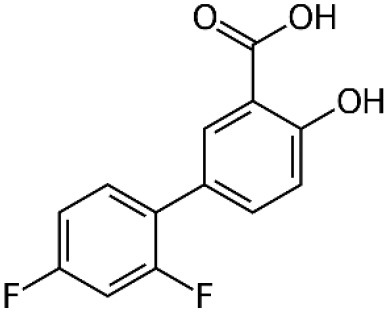	Binding at the thyroxine binding sites/TTR stabilization	Tojo et al., 2006; Berk et al., 2013; Takahashi et al., 2014
**TAFAMIDIS** 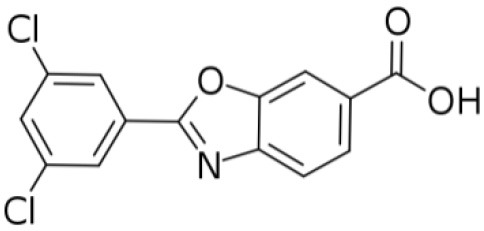	Binding at the thyroxine binding sites/TTR stabilization	Bulawa et al., 2012; Coelho et al., 2016; Barroso et al., 2017; Maurer et al., 2018
**AG10** 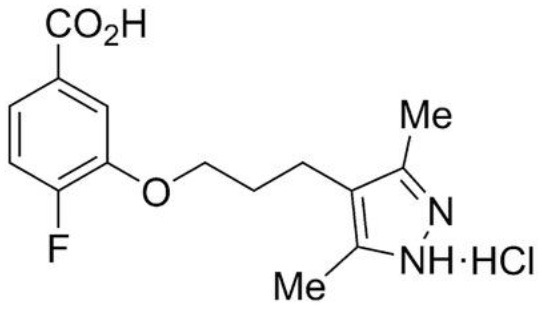	Binding at the thyroxine binding sites/TTR stabilization	Alhamadsheh et al., 2011; Penchala et al., 2013; Miller et al., 2018; Judge et al., 2019
**TOLCAPONE** 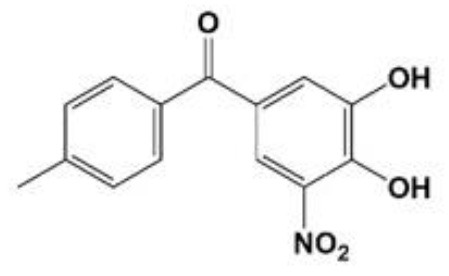	Binding at the thyroxine binding sites/TTR stabilization	Sant'Anna et al., 2016; Gamez et al., 2019; Pinheiro et al., 2020
**mds84** 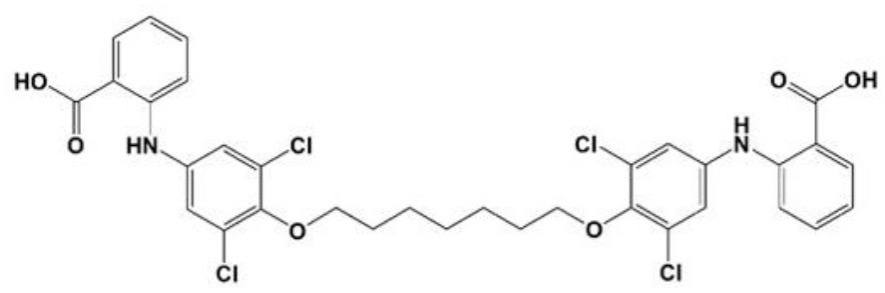	Binding at the thyroxine binding sites-bivalent ligand/TTR stabilization	Kolstoe et al., 2010; Corazza et al., 2019
**CURCUMIN** 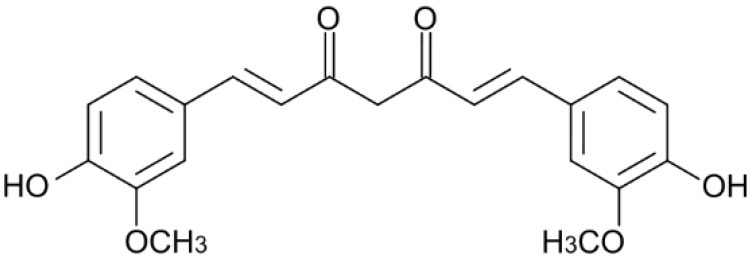	Binding at the thyroxine binding sites/TTR stabilization	Ferreira et al., 2011, 2013, 2016, 2019
**EGCG** 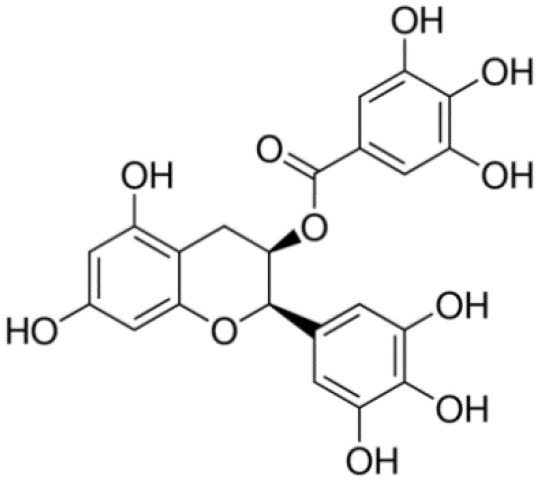	TTR stabilization /inhibition of aggregation (oligomerization)/Disruption of aggregates	Ferreira et al., 2009, 2011, 2012
**Molecular tweezer CLR01** 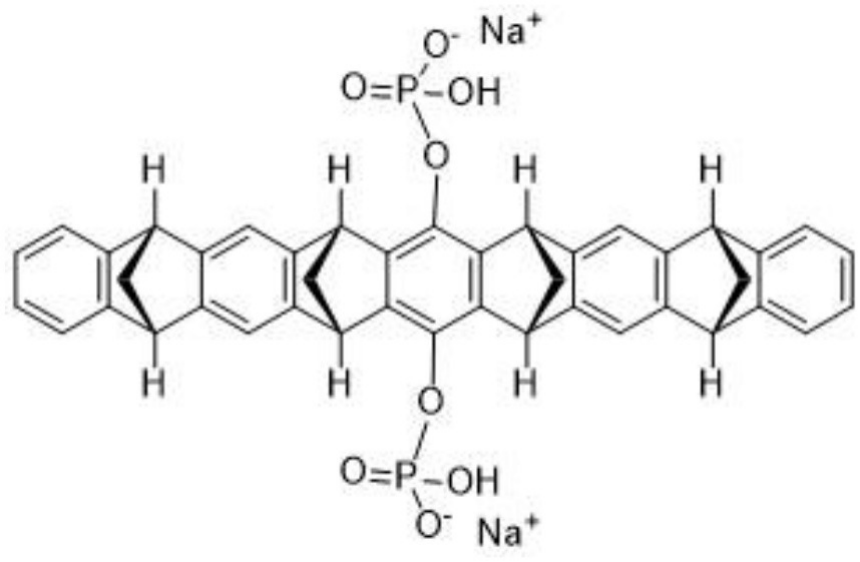	Inhibition of aggregation (oligomerization)/Disruption of aggregates	Sinha et al., 2011; Ferreira et al., 2014
**DOXYCYCLINE** 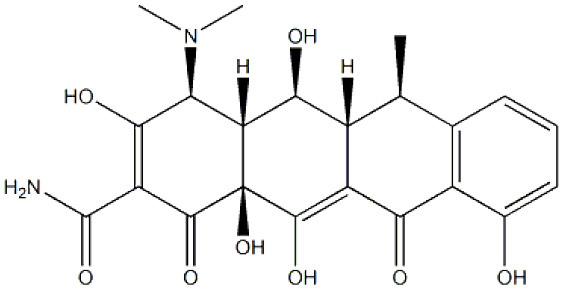	Disruption of TTR aggregates	Cardoso et al., 2003, 2008; Cardoso and Saraiva, 2006

### TTR Stabilization

TTR tetramer stability is a determinant factor conditioning tetramer disassembly, the rate-limiting step for aggregation and amyloid fibrils formation (McCutchen et al., 1993; Quintas et al., 1999). Accordingly, the development of small molecules able to stabilize the TTR tetramer, preventing its dissociation into monomers, has been recognized as a great therapeutic strategy for the treatment of ATTR amyloidosis. The design of these molecules was based on the affinity of T_4_ to bind to the central pocket of the TTR tetramer inhibiting its dissociation (Miroy et al., 1996). Based on the capacity of the nonsteroidal anti-inflammatory drugs (NSAIDs), to bind to the T_4_-binding channel in TTR (Baures et al., 1999; Miller et al., 2004), the first drug to be tested was diflunisal, which was reported as an effective stabilizer of the TTR tetramer in plasmas from ATTR-PN patients (Tojo et al., 2006). Then, ATTRv patients were randomly assigned to receive diflunisal for 2 years and, in fact, the use of diflunisal reduced the rate of progression in neurologic impairment and preserved the quality of life of patients comparatively to placebo group (Berk et al., 2013) and ameliorated the autonomic symptoms in ATTRv patients (Takahashi et al., 2014). However, diflunisal administration to these patients induced long-term side effects, namely impaired renal function and thrombocytopenia (Sekijima et al., 2015), which may compromise its clinical value.

Following, other pharmacologic molecules, such as tafamidis (a benzoaxazole derivative) (Vyndaqel^®^) have been proposed through a structure–based drug design approach to select compounds to occupy these T_4_-binding sites, kinetically stabilizing the TTR tetramer, and ultimately resulting in a decrease in the rate of amyloid fibril formation *in vitro* (Bulawa et al., 2012). One of the major concerns about the use of tafamidis in ATTRv patients is related to potential metabolic side effects, since it could interfere with T_4_ delivery throughout the body. However, clinical trials have found minimal evidences about this concern because thyroxine binding globulin (TBG), rather than TTR, transports the majority of the circulating T_4_ (~75%) (Refetoff, 2000; Coelho et al., 2012). Tafamidis has gained approval for the treatment of ATTRv amyloidosis in several countries, including in the European Union, Mexico, Argentina, Japan and more recently also in the USA for the treatment of ATTR-CM (Coelho et al., 2016). Moreover, an open-label extension study for 6 years also revealed the slowing of neuropathy progression without unexpected adverse effects (Barroso et al., 2017). Recently, the effects of tafamidis on ATTR-CM were also evaluated in both ATTRv and ATTRwt patients in a phase III clinical trial significantly reducing mortality and cardiovascular-related hospitalizations (Maurer et al., 2018).

Most studies for the development of efficient TTR stabilizers were based on rational ligand design and, thus most of the stabilizers are, in general, halogenated biaryl analogs of T_4_, many resembling NSAIDs. However, these molecules act as cyclooxygenase (COX) inhibitors increasing the risk of severe cardiovascular events therefore being contraindicated in patients with ATTR-CM (Mukherjee et al., 2001). Moreover, high-throughput screening studies pointed out a new compound, AG10, as an effective and selective stabilizer of the cardiac TTRwt and TTR V122I protecting human cardiomyocytes from TTR amyloid toxicity (Alhamadsheh et al., 2011; Penchala et al., 2013). Interestingly, structural studies revealed that AG10 is unique in its capacity to form hydrogen bonds with the same serine residues at position 117 that stabilize the non-amyloidogenic TTR T119M variant (Miller et al., 2018). Recent results from phase II clinical trials revealed that AG10 has the potential to be safe and effective for the treatment of ATTR-CM patients either carrying mutant or TTR wt. Phase III clinical trials with AG10 are ongoing (Judge et al., 2019).

In addition, based in its molecular structure, tolcapone, an FDA-approved drug for the treatment of Parkinson's disease, has been repurposed for the treatment of ATTR amyloidosis. Tolcapone specifically binds to TTR in human plasma and, stabilizes the native TTR tetramer *in vivo* in mice and humans. Furthermore, it was also demonstrated that the binding of tolcapone to the recombinants TTR wt and TTR V122I, at the T_4_-binding channel is stronger comparatively to tafamidis (Sant'Anna et al., 2016). These results pointed-out tolcapone as a strong candidate for the treatment of ATTR polyneuropathy and, in fact, it has gained clinical interest and it already passed phase I/II clinical trials (Gamez et al., 2019). A very recent work on the structural characterization of Tolcapone–TTR complexes demonstrates high stabilization and binding affinity of Tolcapone to TTR variants associated with leptomeningeal amyloidosis. These characteristics in association with its ability to cross the blood-brain barrier suggests its particular indication for therapeutic intervention in this type of amyloidosis (Pinheiro et al., 2020).

Contrarily to the above-mentioned compounds, palindromic ligands, such as mds84, rapidly bind simultaneously to both T_4_-binding sites in each tetrameric TTR molecule, which would overcome the problems of negative cooperativity of the binding of the existing drugs, such as tafamidis. Mds84 binds to the native TTR wt in whole serum and, more effectively to the amyloidogenic TTR variants, promoting the stabilization of the TTR tetramer (Kolstoe et al., 2010; Corazza et al., 2019).

Some plant polyphenols which may be part of our diet have also been reported as TTR tetrameric stabilizers. In particular, epigallocatechin-3-gallate (EGCG) and curcumin, the major components of green tea and turmeric, respectively, were able to effectively stabilize the TTR tetramer in human plasmas from both V30M carriers and controls (Ferreira et al., 2011), as well as in plasmas from transgenic mice carrying human TTR V30M variant (HM30 mice) (Ferreira et al., 2012, 2013). It should be noted that these compounds exhibit different ways of action. Curcumin competes with T_4_ for the binding to TTR, meaning that it binds at the T_4_ binding sites, whereas EGCG stabilizes TTR through binding at the surface of the TTR molecule in particular at two binding sites at the dimer-dimer interface exerting an effect similar to a cross-linker. Low bioavailability and low specificity of binding seem to be relevant conditioning factors of their effects *in vivo* in humans (Kristen et al., 2012; aus dem Siepen et al., 2015; Cappelli et al., 2018).

### Inhibition of TTR Aggregation Into Amyloid Fibril

TTR stabilizers, as the above-mentioned small molecule compounds, including EGCG, curcumin, tolcapone and mds84 have also been reported as inhibitors of TTR amyloid formation as consequence of their effect on the first step of TTR aggregation.

Ferreira *et al*. firstly described EGCG as a strong inhibitor of TTR aggregation *in vitro* (Ferreira et al., 2009), by maintaining most of the protein in a non-aggregated soluble form. EGCG also suppressed the amyloid fibril formation pathway in a cell culture system (Ferreira et al., 2009). Later, the role of EGCG on the inhibition of amyloid fibril formation *in vivo* using a well-characterized transgenic murine model of ATTR-PN was also demonstrated. EGCG reduced, in about 50%, the deposition of TTR toxic aggregates in the gastro-intestinal tract and peripheral nervous system (PNS), with a concomitant decrease in the expression of both non-fibrillar-related biomarkers and amyloid deposition markers (Ferreira et al., 2012).

Similar studies using curcumin demonstrated suppression of fibril formation *in vitro* through the generation of small “off-pathway” oligomers (Ferreira et al., 2011) and inhibited this process in transgenic mice carrying human TTR V30M variant. In fact, immunohistochemical analysis of mice tissues revealed that dietary curcumin decreased TTR load in as much as 70% and lowered the cytotoxicity associated with TTR aggregation (Ferreira et al., 2013). Later, it has been shown that dietary curcumin decreases TTR deposition and associated toxicity in the dorsal root ganglia and stomach of aged mice carrying human TTR V30M variant (Ferreira et al., 2016).

Furthermore, synthetic compounds such as tolcapone and mds84 effectively inhibited the process of TTR fibril formation *in vitro* (Kolstoe et al., 2010; Sant'Anna et al., 2016) and, tolcapone was also able to suppress TTR toxicity in cellular models (Sant'Anna et al., 2016).

However, some inhibitors of amyloid formation might act on a different step of the cascade leading to fibril formation that includes, for instance, the polymerization of the intermediate species originating aggregates that evolve to amyloid fibrils. That is the case of the molecular tweezer CLR01 (Sinha et al., 2011). This is a synthetic compound that through binding to positively charged amino acids, in particular lysine and arginine residues in the terminal beta-strands of TTR, inhibit the tight alignment of protofilaments characteristic of amyloid formation. Thus, the molecular tweezer CLR01 inhibited TTR aggregation *in vitro* and also *in vivo* as demonstrated in a study in which TTR V30M mice treated with CLR01 presented decrease of TTR deposition and of associated biomarkers (Ferreira et al., 2014). However, this compound presents limitations related to the low binding affinity to proteins and to its formulation needing improvement of pharmacologic properties.

### Disruption of Aggregates

The role of anthracyclines and, in particular of 4′-iodo-4′-deoxydoxorubicin on the reabsorption of amyloid deposits was related to the almost planar structure of these compounds and the cross β-pleated structure characteristic of all amyloid fibrils (Merlini et al., 1995). Furthermore, doxycycline, a member of tetracycline antibiotics family, structurally homologous to the anthracyclines, was found to be particularly effective on the disruption of TTR amyloid fibrils *in vitro* (Cardoso et al., 2003). In addition, *in vivo* studies on transgenic mice carrying human TTR V30M variant supported the previous *in vitro* findings. Doxycycline was administered to old transgenic mice and, tissue analysis revealed Congo red positive staining only for the non-treated animals from the control group. Additionally, a decrease in several markers associated with TTR amyloid deposition was also reported (Cardoso and Saraiva, 2006; Cardoso et al., 2008). The recent development of doxycycline conjugates, namely polyglutamate-doxycycline, demonstrated an enhanced effect in the clearance of fibrils comparatively to non-conjugated doxycycline only (Conejos-Sanchez et al., 2015).

Since doxycycline has effect only in advanced phases of the amyloidogenic cascade it has been proposed that it could be combined with another drug targeting an earlier phase of the amyloid fibrils assembly (Cardoso et al., 2010). In this sense, tauroursodeoxycholic acid (TUDCA), a hydrophilic biliary acid derivative, gained particular clinical interest for the treatment of ATTR amyloidosis since it has been previously referred to cause a decrease in the deposition of toxic pre-fibrillar TTR oligomers and to reduce the expression of several apoptotic and oxidative biomarkers associated with ATTR amyloid deposition in transgenic murine models treated with TUDCA (Macedo et al., 2008; Cardoso et al., 2010). Clinical trials of combined doxycycline and TUDCA are underway and preliminary results indicate positive effects though more results are necessary to evaluate the impact of this therapeutic approach in disease progression (Obici and Merlini, 2014).

Moreover, some therapeutic compounds are classified as multi-target disease agents, performing a role in different steps of amyloid fibril formation. For instance, compounds such as EGCG and curcumin besides its effects as inhibitors of aggregation act also as disruptors of TTR amyloid deposits. In fact, both natural polyphenols, EGCG and curcumin, efficiently disaggregated pre-formed TTR amyloid fibrils (Ferreira et al., 2011) (Ferreira et al., 2019). Recent studies using transgenic murine models pointed out both curcumin and TUDCA as modulators of cellular autophagy processes, which are involved in the clearance of large protein aggregates (Teixeira et al., 2016).

### Immunotherapy

Immunotherapy is another therapeutic strategy for the treatment of ATTR amyloidosis, which still remains under investigation. Specific antibodies targeting TTR monomers, oligomers or amyloid aggregates may prevent TTR fibrillogenesis. As a first approach, a structure-based strategy was used to develop a TTR conformation-specific antibody targeting pre-fibrillar, misfolded TTR intermediates without recognizing native tetrameric TTR. This is achieved since the antibody (misTTR) targets the residues 89–97 in the polypeptide chain, which are buried in the TTR tetramer, but it is exposed in the monomer, inhibiting fibrillogenesis of misfolded TTR under micromolar concentrations (Galant et al., 2016). This antibody has already entered into phase I clinical trials in ATTRv patients (Macedo et al., 2020).

## Contribution of TTR Proteolysis to Amyloid Formation

Since a long time ago, TTR proteolysis has been suggested to be involved in the mechanisms driving TTR-related amyloidosis (Pitkanen et al., 1984). Therefore, by understanding in detail the molecular mechanisms implicated in the pathophysiology of ATTR amyloidosis, it would be possible to develop new targeted therapies to improve the patients' outcomes.

Several evidences suggest the existence of different types of TTR amyloid fibrils in a range of tissues. In fact, amyloid deposits might be composed by a mixture of both cleaved and full-length TTR (type A) or full-length TTR only (type B). The resulting amyloid deposits are different. Type A fibrils are shorter and exhibit weaker affinity for Congo Red staining than type B fibrils, which are longer, slender and strongly stain with Congo Red (Bergstrom et al., 2005; Ihse et al., 2008, 2011).

Different amyloidogenic fragments may be found in different tissues and could be associated either with ATTRwt or ATTRv amyloidosis (Suhr et al., 2017). Vitreous TTR appeared to be fragmented between the residues Lys48-Thr49, whereas cardiac TTR may be cleaved at multiple sites between the 46–52 amino acid residues in polypeptide chain (Liepnieks et al., 2006). However, peptide 49-127 C-terminal fragment is the main component of *ex vivo* TTR amyloid fibrils in tissue biopsies of cardiac deposits, which is further associated with poor clinical prognosis, often with rapidly progressive cardiac involvement, even after liver transplantation (Gustafsson et al., 2012; Ihse et al., 2013).

The protease responsible for TTR cleavage has not yet been identified. However, the highly specific fragmentation pattern suggests that it could be a trypsin-like serine protease. The three-dimensional structure of this protein region is solvent exposed and potentially accessible for cleavage. In accordance, all amyloidogenic TTR variants showed an increased main chain solvent exposure comparatively to both native and non-amyloidogenic variants, which may result in increased susceptibility to proteolysis (Schormann et al., 1998).

Recent *in vitro* studies, using recombinant trypsin, revealed that the proteolysis/fibrillogenesis pathway is common to several amyloidogenic TTR variants and, the process of cleavage and release of the 49–127 TTR fragment is faster for the highly amyloidogenic variant, TTR S52P, than for the other TTR variants analyzed (Mangione et al., 2014; Marcoux et al., 2015). It requires the action of biomechanical forces provided by sheer stress of physiological fluid flow and, importantly, the non-amyloidogenic TTR T119M is neither cleaved nor generates amyloid fibrils under these conditions. These studies also demonstrated that the TTR stabilizers, mds84, tolcapone, diflunisal and tafamidis, inhibited TTR proteolysis resulting in the inhibition of aggregation. However, the maximum inhibition is only achieved when both T_4_-binding sites in central hydrophobic channel are simultaneously occupied by small ligands (Mangione et al., 2014; Verona et al., 2017). In opposition, natural TTR ligands, T_4_ and RBP, were not able to inhibit TTR cleavage. Nevertheless, binding of RBP, but not T_4_, effectively inhibited the subsequent formation of amyloid fibrils (Mangione et al., 2014).

Due to the exclusive duodenal location of trypsin, it is unlikely that it may contribute to the development of systemic TTR amyloidosis *in vivo*. In *silico* studies recently pointed out plasmin as a plausible pathophysiological candidate protease involved in the process of TTR amyloid formation (Mangione et al., 2018). Furthermore, the ubiquitous distribution of plasmin, its structural similarities to trypsin (Mangione et al., 2018) and the reported activation of plasminogen activation system (PAS) in other amyloid-related disorders, such as Alzheimer's disease (Tucker et al., 2000) and immunoglobulin light chain (AL) amyloidosis (Mumford et al., 2000; Bouma et al., 2007; Uchiba et al., 2009) also indicate that this protease could perform a key role in TTR amyloidogenesis.

Recent studies showed that amorphous protein aggregates are degraded by plasmin, releasing smaller soluble protein fragments, which are cytotoxic *in vitro* for both endothelial and microglial cells (Constantinescu et al., 2017).

Plasmin, similarly to trypsin, selectively cleaves TTR S52P variant, at Lys48-Thr49 peptide bond under physiological conditions *in vitro* being, both the TTR fragments and full-length protomers readily released from the homotetramer and incorporated into amyloid fibrils, morphologically identical to *ex vivo* TTR amyloid (Mangione et al., 2018). Concerning these observations, a hypothetical model for the role of plasmin-mediated proteolysis on TTR fibrillogenesis has been proposed. In this model, circulating TTR can diffuse toward the extracellular compartment, be entrapped in the fibrin clot or escape from it. Upon plasminogen activation, TTR may be cleaved and then dissociate into a mixture of both truncated and full-length TTR, which ultimately assemble into amyloid fibrils and deposit at the extracellular space (Mangione et al., 2018).

Altogether these evidences seem to point out the importance of lysine (Lys) residues for the pathogenicity of ATTR amyloidosis as it has been described for other amyloid disorders (Sinha et al., 2011). By targeting the Lys residues using synthetic Lys specific molecular tweezers (e.g., CLR01), the process of TTR proteolysis could be effectively inhibited through its binding to Lys48, which seem to be target of the protease responsible for TTR cleavage. This could be particularly important for the treatment of both ATTR-CM and vitreous amyloidosis, since the 49-127 TTR fragment has been frequently encountered in the amyloid deposits in both cases.

Despite the increasing interest on TTR proteolysis as leading mechanism-driving ATTR amyloidosis, some questions remain to be answered. Though, it is still unknown whether TTR fragmentation occurs prior or after aggregation and, where it occurs, in circulation or at the site of deposition, an increase of the proteolytic activity in plasmas from ATTR patients comparatively to healthy controls, suggesting that the process occurs in the bloodstream before fibril formation (da Costa et al., 2015).

## Extracellular Chaperones as Regulators of ATTR Amyloidosis

The disruption of the protein folding quality control mechanisms is also an underlying cause of ATTR amyloidosis. Recently, some studies revealed the existence of a growing family of extracellular chaperones in body fluids, which selectively bind to exposed hydrophobic residues in misfolded proteins in order to prevent their toxicity upon aggregation into insoluble deposits (Wyatt et al., 2013).

Among those extracellular chaperones, haptoglobin, alpha-2-microglobulin (A2M) and clusterin were found to be increased in plasmas from ATTR patients (da Costa et al., 2015). While haptoglobin and A2M, were previously described as effective in the inhibition of stress-induced aggregation of a number of unrelated target proteins (Yerbury et al., 2005; French et al., 2008), clusterin is an ubiquitous highly conserved secreted protein (Wyatt et al., 2009), which inhibits protein aggregation in an ATP-independent manner upon its binding to misfolded proteins, such as α-synuclein and β-amyloid peptide, producing soluble, high molecular complexes (Matsubara et al., 1996; Poon et al., 2000; Yerbury et al., 2007).

The role of clusterin on the clearance of extracellular aggregates has also been investigated in ATTR-PN (Lee et al., 2009; Magalhaes and Saraiva, 2011). *In vitro* studies using neuroblastoma cells incubated with TTR oligomers revealed intracellular clusterin overexpression and increased levels of clusterin secreted to the culture medium. An overexpression of clusterin in tissues with TTR deposition was found in mice carrying human TTR V30M in HSF-1 null background, which exhibit early and extensive non-fibrillar TTR deposition in the gastrointestinal tract and in the peripheral and autonomic nervous system. In addition, in human nerve, clusterin co-localizes either with fibrillar or non-fibrillar TTR deposits as detected by double immunostaining (Magalhaes and Saraiva, 2011).

Clusterin was also found in cardiac TTR amyloid deposits from patients with ATTRwt and ATTRv (Greene et al., 2011) and, later, experiments using circular dichroism spectroscopy revealed that clusterin preferentially stabilizes monomeric TTR leading to the appearance of increasingly stable conformations under acid stress. Additionaly, clusterin interacts also with high molecular weight TTR aggregated species and, these interactions with both monomeric and oligomeric TTR proceed in a cooperative manner in the presence of the TTR tetramer stabilizer, diflunisal. Altogether these observations suggest a novel synergistic treatment for ATTR amyloidosis using both diflunisal and clusterin for the removal of misfolded and aggregated TTR (Greene et al., 2015). Accordingly, preliminary data revealed a temporal increase in serum clusterin levels in patients treated with diflunisal at 1-year follow-up compared to baseline. In opposition, patients who were not treated with diflunisal demonstrated decreased clusterin levels at annual evaluation. Interestingly, a positive correlation between clusterin and TTR levels was found at baseline suggesting that soluble tetrameric TTR decreases as more of the native protein dissociates and forms species, overwhelming the protein folding capacity of clusterin leading to a reduction in circulating levels of this molecular chaperone and, the treatment of the ATTR patients with diflunisal lead to a partial recovery of serum clusterin levels (Torres-Arancivia and Connors, 2019). These results are in accordance with previous studies reporting the beneficial effects of diflunisal for the treatment of ATTRv amyloidosis.

## Conclusion and Future Perspectives

ATTR amyloidosis is an under-recognized disease which is characterized by extracellular deposition of TTR aggregates in several organs, being polyneuropathy and cardiomyopathy the major clinical manifestations. The mechanism by which the tetramer disassembles and aggregates into amyloid fibrils has been considered the main driver of the disease. However, TTR proteolysis, namely occurring in the cardiac tissue, as well as its modulation have been increasingly documented as fundamental for understanding the development and progression of ATTR amyloidosis.

**Figure 2:**
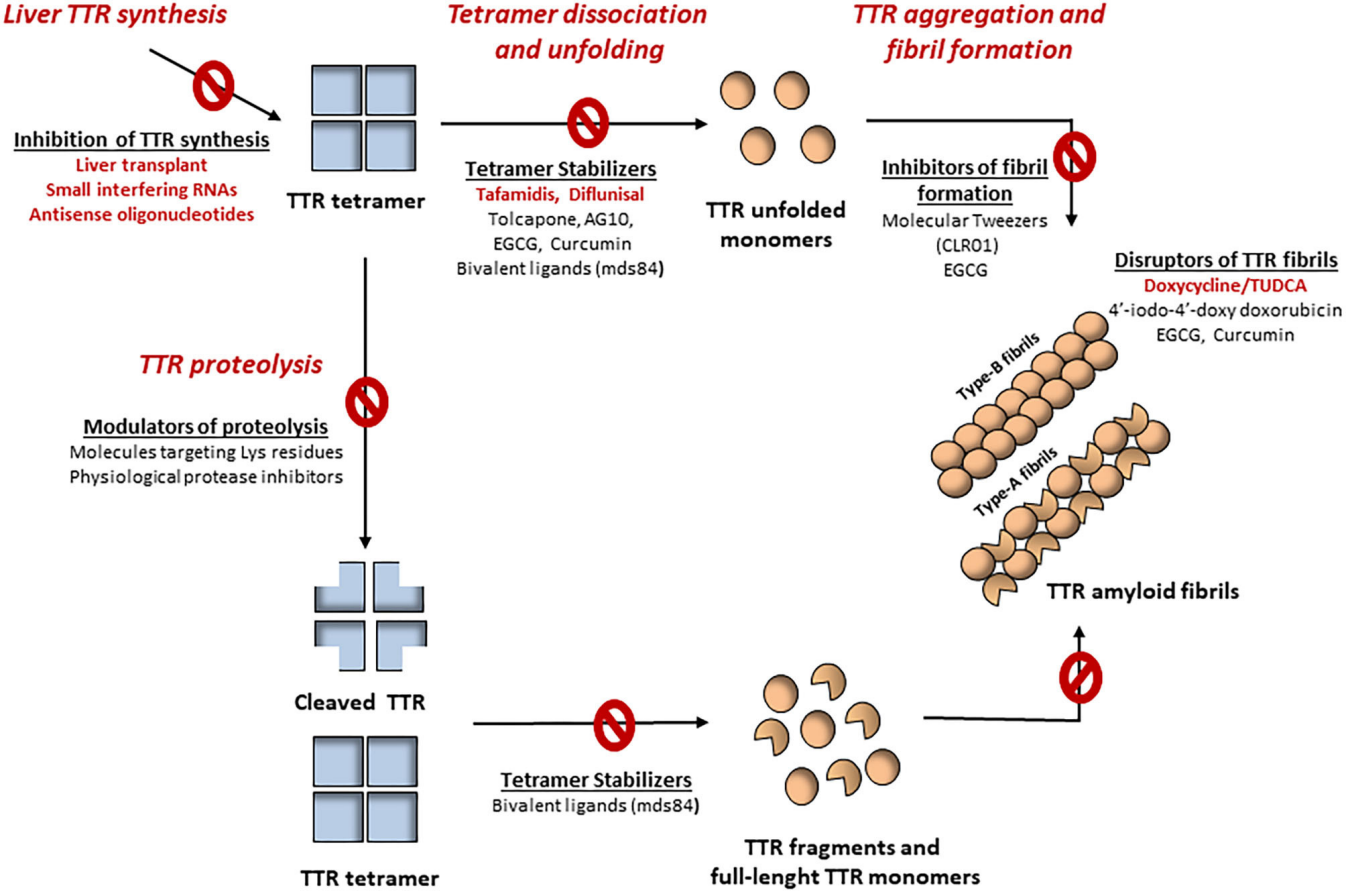
Mechanisms-driving TTR amyloidogenesis and different therapeutic targets for the treatment of ATTR amyloidosis. Tetramer destabilization is widely accepted as a rate-limiting step for the development of amyloid fibrils. However, TTR proteolysis has been increasingly suggested as an alternative mechanism contributing to amyloid formation. Several pharmacological agents have been implicated in the treatment of ATTR amyloidosis, from inhibitors of TTR synthesis, tetramer stabilizers, inhibitors of amyloid formation and even disruptors of formed fibrils. The modulation of TTR proteolysis may also be helpful for the treatment of ATTR amyloidosis. Despite the protease responsible for this process has not yet been identified, its specific cleavage patterns suggest that it could be a trypsin-like serine protease. Accordingly, pharmacological molecules targeting lysine residues, as well as physiological serine protease inhibitors may be act as modulators of TTR proteolysis, consequently inhibiting amyloid formation.

Many therapeutic approaches have been suggested for the treatment of ATTR amyloidosis targeting different steps of the pathology. Those therapies include interventions from the synthesis of the TTR variants through liver transplant or gene silencing therapies, to TTR stabilization, inhibition of aggregation, disruption of amyloid fibrils and clearance of amyloid deposits. The main targets for intervention on TTR amyloid formation are summarized in Figure 2. Although some the available therapies are more efficient than others, it becomes increasingly evident that combination of different therapies may improve the therapeutic outcome. In this sense, it would be interesting to test TTR gene silencing therapies in combination with protein stabilizers or disruptors of pre-existing amyloid deposits. It is also important to obtain more efficient and targeted therapies specific to organ and tissues with limited drug access as is the case of the eye and brain, that are particularly relevant in some forms of the disease. Moreover, it is crucial to continue with studies that can contribute to a better understanding of the mechanisms involved in the disease, in particular, TTR proteolysis, which has been mainly valued in the case of ATTR-CM and, also at the extracellular level involving either interactions with components of the extracellular matrix or with molecular and chemical chaperones acting as disease modulators.

Overall, detailed knowledge of the mechanisms of amyloid formation and the availability of different approaches allows directed and personalized interventions aiming higher specificity and efficacy of chosen therapeutic solutions.

## Author Contributions

FB, MS, and MA wrote and discussed the manuscript. All authors contributed to the article and approved the submitted version.

## Conflict of Interest

The authors declare that the research was conducted in the absence of any commercial or financial relationships that could be construed as a potential conflict of interest.
